# Rational Design of Bioactive Materials for Bone Hemostasis and Defect Repair

**DOI:** 10.34133/cbsystems.0058

**Published:** 2023-10-11

**Authors:** Yuqi Gai, Yue Yin, Ling Guan, Shengchang Zhang, Jiatian Chen, Junyuan Yang, Huaijuan Zhou, Jinhua Li

**Affiliations:** ^1^ School of Medical Technology, Beijing Institute of Technology, Beijing 100081, China.; ^2^ Advanced Research Institute of Multidisciplinary Sciences, Beijing Institute of Technology, Beijing, 100081, China.; ^3^ Department of Medicine, University of British Columbia, Vancouver, BC, Canada.; ^4^ National Center for Neurological Disorders, Beijing Tiantan Hospital, Capital Medical University, Beijing 100070, China.

## Abstract

Everyday unnatural events such as trauma, accidents, military conflict, disasters, and even medical malpractice create open wounds and massive blood loss, which can be life-threatening. Fractures and large bone defects are among the most common types of injuries. Traditional treatment methods usually involve rapid hemostasis and wound closure, which are convenient and fast but may result in various complications such as nerve injury, deep infection, vascular injury, and deep hematomas. To address these complications, various studies have been conducted on new materials that can be degraded in the body and reduce inflammation and abscesses in the surgical area. This review presents the latest research progress in biomaterials for bone hemostasis and repair. The mechanisms of bone hemostasis and bone healing are first introduced and then principles for rational design of biomaterials are summarized. After providing representative examples of hemostatic biomaterials for bone repair, future challenges and opportunities in the field are proposed.

## Introduction

**Fig. 1.**
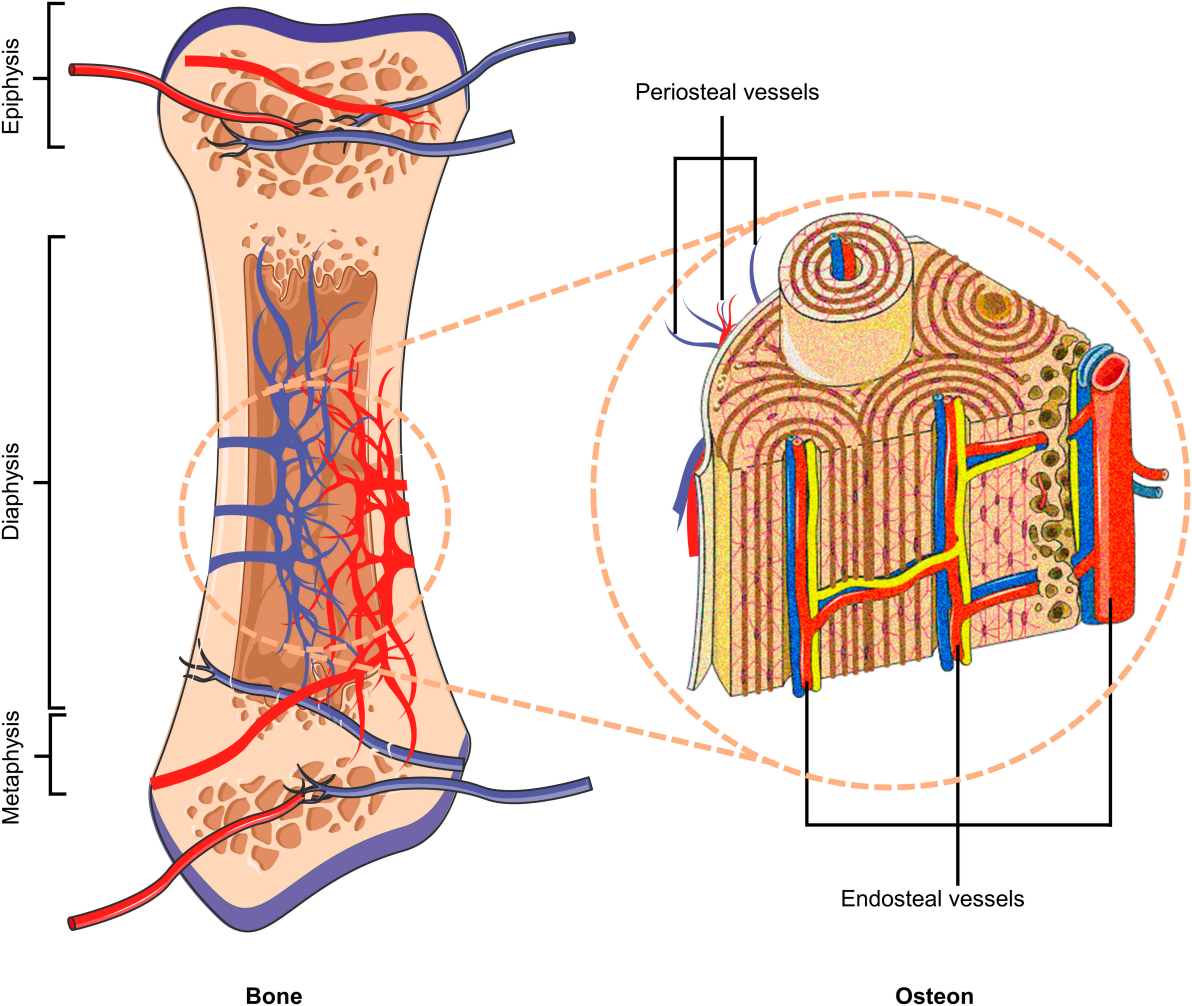
Schematic diagram of the blood supply system from a long bone: Different blood supply modes are presented in the backbone, metaphyseal, and epiphysis. To maintain normal blood circulation, the blood exchange in the bone is achieved by connecting with the surrounding tissue structures such as the periosteum, muscles, and joints.

As the basic skeleton of the human body, bone is a connective tissue that combines hardness and toughness with a high degree of functionalization. The main function of bone is to support the body, protect organs, store minerals, and offer hematopoiesis [1,2]. Bone tissue is not limited to bone; it also includes blood vessels, periosteum, bone marrow, and lymphatic vessels [3–6]. Bone tissue is a vascularized tissue, as shown in Fig. 1 [6]. The self-healing potential of bone tissue is limited. When bone tissue is slightly damaged, it can stop bleeding and heal on its own. When faced with severe open trauma and critical surgical situations that may lead to large bone defects, the bone tissue itself cannot stop bleeding in time. In such cases, external measures must be urgently used to stop the bleeding since uncontrollable bleeding directly affects associated morbidity and mortality.

Currently, most traditional surgical hemostatic treatments for surgical and post-traumatic wounds firstly use absorbable agents to compress open wounds and then use tissue adhesives to seal the wounds [7,8]. For open bone trauma, hemostatic agents are utilized to quickly stop bleeding. However, bone hemostasis (especially in terms of wounds with variable shapes and deep positions) still faces many challenges, such as difficulty in timely bleeding, susceptibility to infection, and unsatisfactory hemostatic efficacy. Taking bone wax as an example, it is a classic hemostatic material for treating bone hemorrhage in orthopedic surgery for nearly a century due to its excellent hemostatic capacity, ease of use, and low cost [9,10]. However, clinical case reports suggest that the use of traditional bone wax carries a risk of causing allergic reactions and foreign body rejection at the surgical site [11–13]. Most importantly, the low biodegradability of traditional bone wax seriously affects the regeneration of bone defects. In the face of complex environments (e.g., field battles), hemostatic gauze [14] and sponge [15] can promptly play a role in treating wounds. However, hemostasis through external pressure and absorption will have some impacts on the bleeding site, and at the same time, it also faces problems such as excessive plasma absorption and low success rate of hemostasis, and it affects healing of the affected area. Hence, understanding the mechanism of bone hemostasis and bone repair is a prerequisite for the development of efficient bone hemostatic biomaterials.

After the investigation of various natural and synthetic biomaterials, surgeons can obtain a wide range of hemostatic materials (e.g., hydrogel [16], robust bandage [17], bioadhesive [18], bio-glue [19], and hemostatic powder [20]) to treat externally palpable injuries. Although some reviews have provided valuable guidance for designing hemostatic materials and scaffold materials [21–24], comprehensive studies focusing on bone hemostasis and bone repair are still absent. The research on hemostatic materials is still in the developing stage, and there is still a long way to go before commercial use. It is of desperate importance to develop novel functional materials or traditional materials after modifications to accelerate bone hemostasis and repair bone defects.

This review mainly summarizes the latest research advancements in biomaterials for bone hemostasis and repair. It covers the design principles, optimization methods, current applications, and future directions of these biomaterials. Starting from addressing bone bleeding and hard-to-healing, the paper first introduces the principles of bone healing and the basic mechanisms of biohemostatic materials. After discussing the scientific design of hemostatic materials for bone repair, representative bone repair hemostatic biomaterials are presented. Finally, the challenges and future research directions in the field of biohydrogels for bone healing are discussed.

## Mechanism of Hemostasis and Healing

As a Chinese saying goes, it usually takes around 100 days to recover from a bone fracture/injury. It can be seen that the healing process for injured bone tissue is relatively long. Hemostasis is one indispensable step in the healing process, followed by a series of processes such as inflammatory response and tissue proliferation [22].

### Mechanism of bone hemostasis

**Fig. 2.**
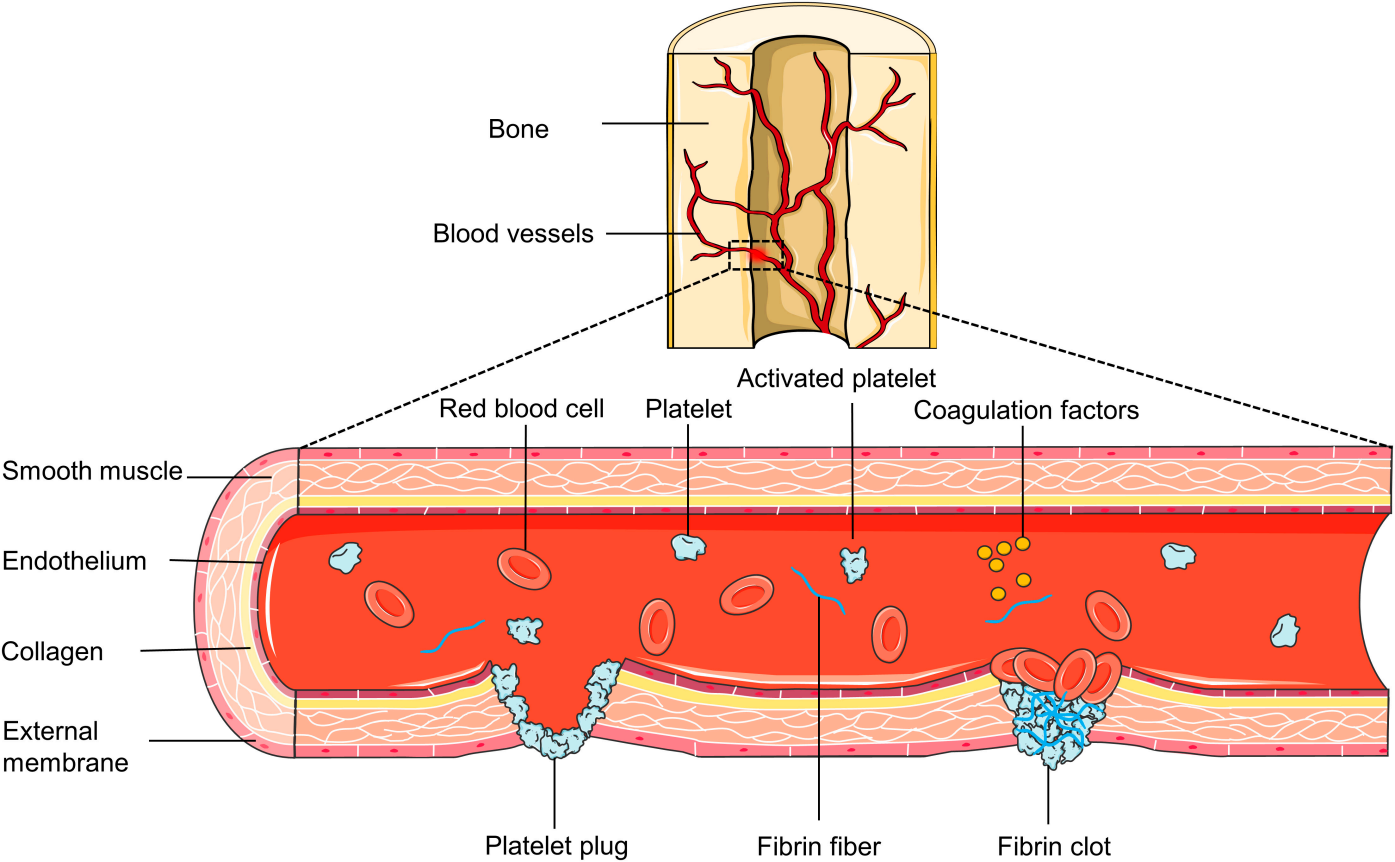
Schematic diagram of bone hemostasis process. For damaged bone tissue, blood flows out of blood vessels and, at the same time, blood cells accumulate at the wound site. As part of the accumulated platelets are activated, a coagulation process starts. Finally, a blood cell embolism is formed to block the wound, preventing the bleeding.

In each tissue, the process of hemostasis reaction is almost the same. After acute trauma, local blood vessels at the wound rupture and bleed. Immediately, the blood begins to coagulate by itself. At this stage, blood cells aggregate around the wound to form a coagulation clot, which can effectively prevent the injury caused by continued bleeding from the wound [25,26]. The formation of a stable hemostatic state at the site of vascular injury requires a series of complex procedures [27]. As shown in Fig. 2, the initiation of primary clotting reactions is triggered when the injury occurs. As a result, vasoconstriction occurs around the wound to slow down blood flow and the endothelium is exposed, making platelets possess enough time to adhere and be activated at the site of injury. As the activated platelets attract and activate adjacent platelets, a temporary platelet plug is formed to seal the wound. Over time, secondary clotting reactions gradually come into effect. After completing a complex cascade with the involvement of clotting factors, collagen fibers, platelets, and Ca^2+^, the platelet plug is transformed into a more stable fibrin clot, and eventually a scab is formed [25,28]. In bone tissue, a notable characteristic of the hemostatic response is the generation of a large and distinct blood clot between the ends of a fractured bone, which is referred to as a “hematoma” [29].

### Mechanism of bone healing

Bone tissue is a vascularized tissue, as shown in Fig. 1. The healing process of bone tissue is similar to that of ordinary wounds. However, certain bone tissues have specific repair processes [30,31]. When a bone injury occurs, the periosteum, endosteum, and soft tissue blood vessels around the bone will be immediately damaged. Bleeding and edema happen in the injury site, causing insufficient oxygen supply and nutrient supply of local bone cells, which leads to necrosis. At the same time, the healing process in the damaged area is being carried out simultaneously.

**Fig. 3.**
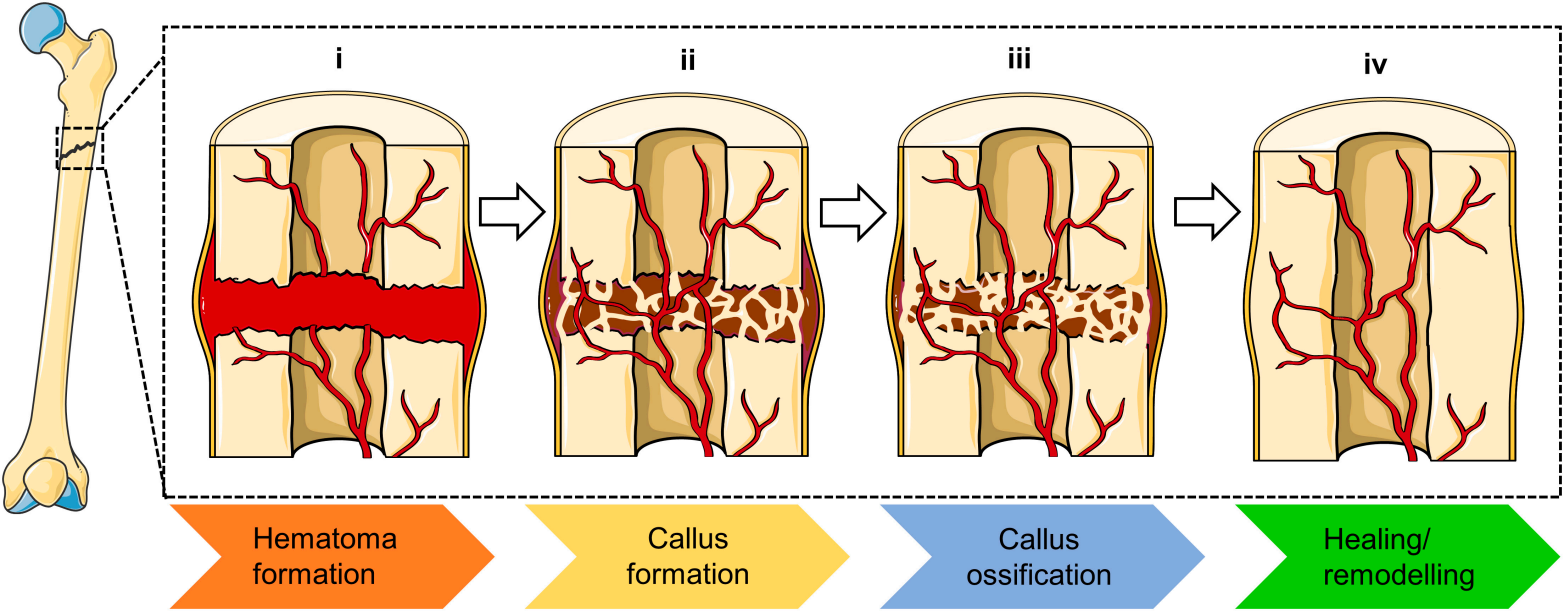
Schematic diagram of 4 bone healing stages.

As demonstrated in Fig. 3, the bone healing process can be broadly divided into 4 stages: hematoma formation, callus tissue formation, callus tissue ossification, and bone tissue healing/remodeling, which is somewhat different from the normal wound healing process [32,33]. In the first stage, the bone tissue successfully stops bleeding after injury, forming a hematoma. The hematoma structure is formed to maintain the integrity of blood vessels in bone tissue. Most importantly, it can stabilize the terminal state of a broken bone, help bone tissue achieve remodeling, and promote inflammatory response and granulation tissue formation. In the second stage, the fibrin network as a natural tissue-engineered scaffold provides an environment for cell adhesion and proliferation in hematoma [29]. Fibroblasts replicate in large quantities and produce collagen to fill wounds, and immune cells, bone tissue cells, and blood cells can be fully fixed on the fibrin web, promoting active healing of bone tissue. The trapped erythrocytes and leukocytes in the fibrin network activate platelets for degranulation, attracting pro-healing cells such as monocytes, fibroblasts, and osteoblasts from the periosteum of fractures. They move to the wound, promoting osteoblast cell differentiation and further growth [34]. In the third stage, the new bone tissue gradually replaces the original collagen fiber network and gradually calcifies. Some proteins secreted by cells can promote cell attachment and migration to fibrin, control the gradual degradation and replacement of blood clots, and promote bone regeneration. This process transforms the blood scab into a harder fibrocartilage callus, which can join broken bones together. In the fourth stage, bone tissue is mainly reshaped and woven into new bone under the dual action of osteoblasts and osteoclasts [35,36]. Therefore, it will take a long time for the bone tissue to return to its primitive state of basic health.

The cause of bone healing is not singular, and it is a highly complex process. For example, the hematoma structures can affect the migration of bone tissue remodeling cells, angiogenesis, and final osteogenesis by releasing growth factors such as bone morphogenetic protein [37,38]. To date, however, no material performs better than autologous bone grafting in terms of bone-forming [2]. Therefore, the screening and update of biomaterials are particularly important.

### Hemostatic biomaterials

For hemostatic biological materials, the hemostatic mechanism is mainly achieved by the corresponding physical and chemical reactions between the applied hemostatic materials and the biomolecules related to the coagulation process in the body. The hemostatic mechanism can be roughly divided into physical hemostasis, chemical hemostasis, and physiological hemostasis. Physical hemostasis using methods such as tourniquet, balloon tamponade, packing, and the antishock garment is achieved by quickly and safely closing the wound before a clotting reaction occurs [39]. Chemical hemostasis and physiological hemostasis mainly affect the primary coagulation process and the secondary coagulation process of wound healing [40]. They participate in the body’s coagulation reaction in an active way, such as accelerating and amplifying the effect of the coagulation cascade to promote the body’s coagulation. Furthermore, they can activate corresponding coagulation factors, so that the coagulation process can reach a certain stage and even accelerate coagulation, where blood cells accumulate at the wound to facilitate wound clotting.

The adhesion and aggregation of platelets in the wound can be guided by utilizing materials with specific physiochemical characteristics, such as hydrophilic or hydrophobic surface, 3-dimensional (3D) structure [41], and surface charges [42]. For instance, the increase of negative charge density on the surface of materials can enhance the activity of platelets while materials with positively charged surfaces can attract negatively charged platelets and facilitate platelet adhesion [42,43]. Although directly activating coagulation factors can effectively prevent the wound from bleeding, potential systemic thrombosis cannot be ignored as coagulation is released into the blood. In contrast, the indirect activation of coagulation factors by using surface-functionalized materials to achieve wound coagulation is more valuable in hemostatic applications. For example, protein-based hemostatic biomaterials (e.g., fibrin glue, collagen, and gelatin) can be used to bind platelets or red blood cells to activate clotting factors, and ultimately participate in coagulation [44].

The hemostatic materials can passively plug the wound in a simple mechanical way, thus avoiding the generation of a thrombus mass and promoting rapid recovery. Hydrogels have been widely used as hemostatic materials as they can perfectly close the wound [45,46]. Since hydrogel can absorb plasma during swelling, the unit concentration of red blood cells and clotting factors becomes higher, which can accelerate the formation of blood clots. Plasma absorption is also the basic mechanism for many inorganic materials (e.g., graphene-based sponges [47] and electrospun nanoclay membranes [17]) and those with surface charges [48]. However, excessive absorption of blood will lead to massive blood loss and subsequent complications. Hence, researchers have attempted to design hemostatic materials by choosing suitable surface wetting properties (e.g., superhydrophobic hemostatic nanofiber [49], hydrophilic chitosan (CS)/graphene oxide composite sponge [50], and cotton gauze with a controlled balance of hydrophilicity and hydrophobicity [51]) to avoid tragedy. Since the inflammatory reaction at the wound becomes more intense due to the infected environment, it is of great urgency to inhibit the concentration of harmful bacteria in the wound and simultaneously promote wound healing. Hence, many researchers have focused on the design and fabrication of antibacterial hemostatic materials [52,53].

## Design Considerations of Hemostatic Materials for Bone Repair

Materials in various forms (e.g., powders, adhesives, hydrogels, injections, and sealants) have been adopted in bone hemostasis and bone repair. Taking hydrogel-based hemostatic material as an example, we will briefly describe the fundamental material design principles. Hydrogel is a 3D polymer network composed of hydrophilic polymer chains, which is soft and absorbable. It behaves like natural extracellular matrices, providing an environment suitable for endogenous cell growth. Due to its 3D network structure, the hydrogel can encapsulate proteins, cells, and drugs in its pores. In addition, a hydrogel can be designed in any required geometrical shape, providing a platform for the personalized repair of bone defects with irregular shapes. However, low mechanical strength is the main barrier hindering its practical application. Researchers have attempted various approaches to enhance its mechanical properties [54–56]. To develop hemostatic materials for bone tissue engineering, the main design principles can be summarized as follows:

1. Bone hemostatic and repair ability: The varying charges on the surface of materials have positive effects on hemostasis. Specifically, materials with cationic charges can attract platelets to aggregate and promote hemostasis, while materials with anionic charges can sufficiently stimulate the hemostatic activity of platelets to promote hemostasis [57,58]. It is safer and more efficient to modify the surface signal of decorative materials to regulate the coagulation mechanism or directly load the modified signal molecules and drugs into the material to prevent bleeding. Among them, the charge of hydrogel materials can be flexibly regulated, and they can absorb plasma, separate blood cells, promote blood coagulation, and regulate platelet activity to accelerate hemostasis [59,60]. However, the general hydrogel materials may swell in vivo, which causes interaction with the deep tissue in vivo, other side effects, and discomfort; therefore, it is necessary to develop anti-swelling hydrogels or hydrogels with strong mechanical strength.

Hydrogel materials have a regular structure with a 3D micro-network, which is beneficial for cells to enter and grow in the hydrogel. The tissue can be sufficiently vascularized and directly generate bone in case of the large pore size of the hydrogel materials, while the neoangiogenesis can be reduced and the cartilage can be formed into bone in case of the small pore size of the hydrogel materials. The regeneration pathway of bone tissue cells can be changed by adjusting the pore size and porosity of the material. Generally, pure hydrogel materials have poor mechanical strength, and bone tissue regeneration requires certain stress stimulation. Therefore, composite materials can be selected and investigated to enhance mechanical strength [61,62].

2. Bone infection therapy: Although the hydrogel materials could play a positive role in both rapid hemostasis and healing of large bone defects, it can easily cause tissue infection, aseptic inflammation, and other immune-related adverse events such as foreign bodies, which are supposed to be dissolved urgently [63]. It is reported that recently developed materials can directly access the intrinsic properties of antimicrobial properties. Specifically, hydrogel materials loaded with metal ions generally obtain antibacterial capacities, while materials with positive charges on the surface can be adopted to limit the activities of bacteria with negative charges on the surface through electrostatic interaction, thereby eventually leading to apoptosis of bacteria. In addition, antibiotics or other drugs with anti-inflammatory effects can also be directly loaded into the hydrogel material for an efficient therapeutic effect [64,65].

3. Other demands of bone therapy: As traditional bone repair materials fail to meet the growing clinical requirements, scientists have paid attention to developing novel biomaterials, especially hydrogel-based materials. The porous 3D network of hydrogel systems can provide a suitable platform for cell spreading and proliferation. Most importantly, some hydrogels have intrinsic bioactivities, such as antibacterial performance [66], osteogenesis capacity [67], and hemostasis [57]. Taking tooth extraction as an example, traditional treatment methods mainly pay attention to hemostasis after tooth extraction, neglecting problems such as potential defects and resorption of alveolar bone. New materials, however, can stop bleeding while protecting the alveolar bone and promoting bone regeneration [68]. Basically, biomaterials are supposed to be biodegradable and biocompatible, so that these materials do not cause cytotoxicity or other side effects to humans and can be completely decomposed after accomplishing the task of tissue repair [69]. In traditional orthopedic surgery, the residue of bone wax can lead to many complications. Hence, new bone wax has been developed to achieve both absorbable and hemostatic effects, which have a positive effect on tissue repair [70].

The incorporation of angiogenic factors into biomaterials has emerged as an effective strategy to enhance bone repair [71]. In the process of bone healing, various cells and factors have been involved in tissue repair. The representative angiogenic factors include vascular endothelial cell growth factor, fibroblast growth factor, bone morphogenetic protein, and transforming growth factor, and each factor has a specific functionality [72]. By artificially increasing the proportion of a specific growth factor in the wound, such as embedding angiogenesis factors into hydrogels, the therapy efficiency of bone repair can be largely enhanced [73,74]. It has been confirmed that vascularization and bone regeneration during bone repair can be promoted by loading vascular endothelial growth factor and bone morphogenetic protein 2 in hydrogels [75]. It is noteworthy to mention that the immune response may be excessive when the concentration of the factor in the local area is extremely high. When the factor is added directly through exogenous sources, however, the utilization efficiency of the factor may be extremely low and most factors may be decomposed [76].

## Hemostatic Biomaterials for Bone Repair Applications

### Biopolymers

**Fig. 4.**
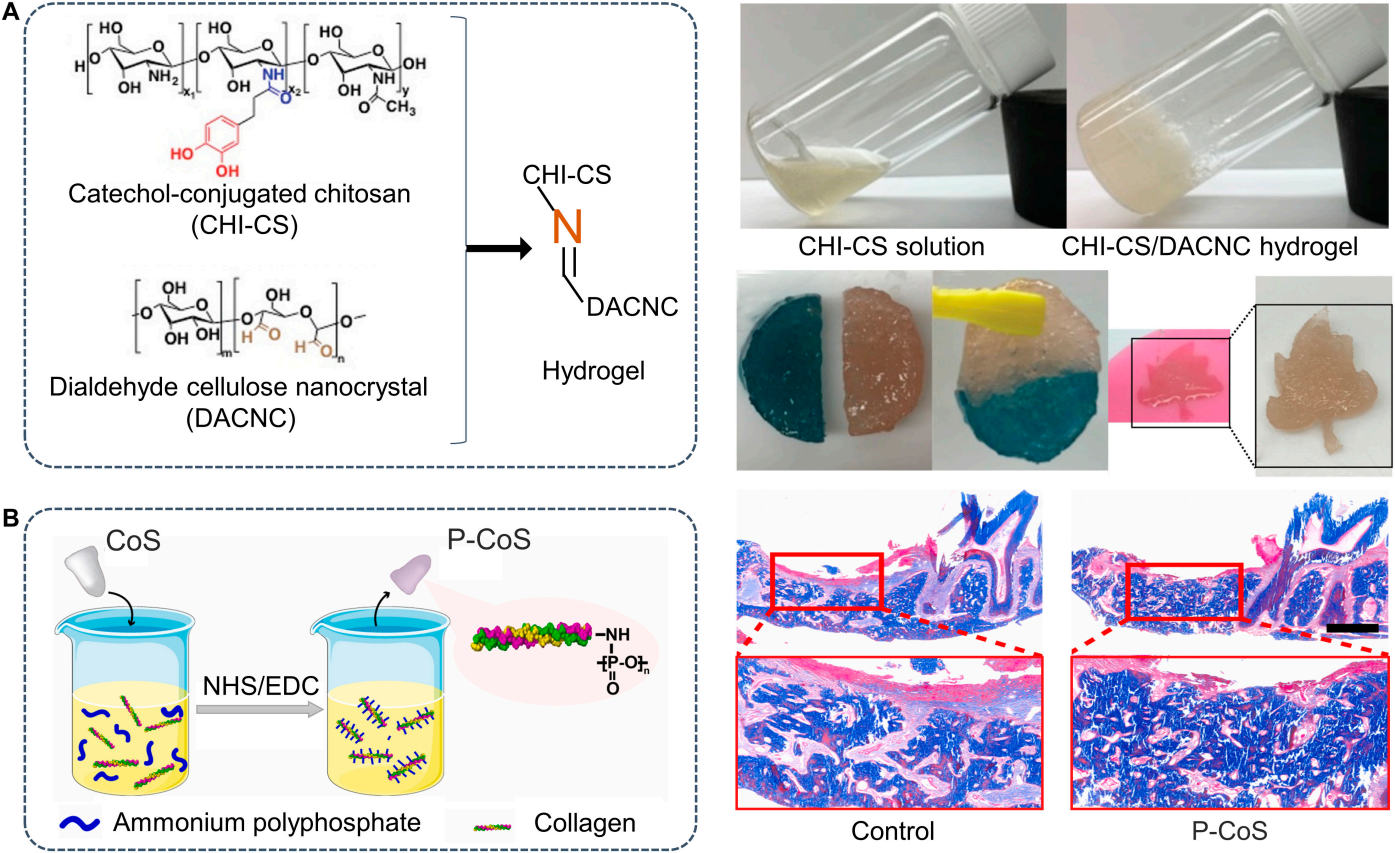
Biopolymer-based hemostatic materials for bone repair. (A) Synthesis of CHI-CS/DACNC hydrogel and fast cross-linking of CHI-CS with DACNC to form a hydrogel, which has the self-healing property. Reproduced with permission from Ref. [79]. Copyright 2021 Wiley-VCH GmbH. (B) Synthesis of polyphosphate onto a CoS (left). MT staining of rat alveolar bone defect model after 3 weeks (right). Reproduced with permission from Ref. [68]. Copyright 2021 The Authors.

Biopolymers are also referred to as natural polymers [4]. Polysaccharides, typical biopolymers, are formed by the dehydration of multiple monosaccharide molecules and joined by the glycosidic bond. They are widely distributed in nature. As a unique natural polysaccharide, CS is a commonly used hemostatic material. When in contact with blood, the surface of CS becomes protonated by the presence of free amino groups (-NH_2_), forming -NH_3_^+^. This process stimulates the adhesion and aggregation of platelets and red blood cells, resulting in the formation of a thrombus [77,78]. Due to its hemostatic property, various hemostatic materials have been developed based on CS. Huang et al. [79] synthesized a multifunctional self-healing hydrogel by cross-linking catechol-conjugated chitosan (CHI-CS) with aldehyde-modified cellulose nanocrystals (DACNC), as shown in Fig. 4A. This hydrogel not only exhibited good adhesion properties to the tissue under moist conditions but also excellent hemostatic performance and biodegradability. Other studies also confirmed that CS-based biomaterials exhibited a superior hemostatic effect and the effect of promoting bone regeneration [80,81]. Hyaluronic acid (HA) is widely found in living organisms and is a natural glycosaminoglycan. Increased HA concentration at the wound site can promote the migration of inflammatory cells and fibroblasts to the wound, which is conducive to wound repair. Kim et al. [82] used negatively charged HA and positively charged recombinant mussel adhesion protein (rMAP) to develop an rMAP/HA agglomerate bone graft adhesive hydrogel, which has enhanced mechanical properties, hemostatic properties, and the ability to repair large bone defects. As shown in Fig. 4B, Gu et al. [68] designed a polyphosphate cross-linked collagen scaffold (CoS), which can not only promote hemostasis and alveolar bone regeneration in the alveolar bone area after tooth extraction but also protect the alveolar ridge. Furthermore, this CoS can be easily degraded.

In addition, many other polysaccharides have been used as hemostatic materials. As one of the most common natural polymer biomaterials, alginate can be cross-linked by metal ions (e.g., calcium ions and sodium ions) to form hydrogels. Among them, calcium ions themselves can promote a coagulation reaction [83]. Cellulose and starch both have low prices and unique liquid absorption characteristics. Through surface functionalization or combination with other materials, they can promote the regeneration of new bone tissue and osteoblastic tissue differentiation [84–88]. Sacchachitin (SC), extracted from *Ganoderma lucidum*, can also be used to stop bleeding. SC nanofibers can be oxidized by 2,2,6,6-tetramethyl-1-piperidinyloxy to form a 3D gel scaffold, which has a good pro-healing effect on rat femoral defects [89]. Both peptides and proteins are composed of amino acids with different charges and hydrophilicity, allowing them to interact physically and chemically with blood cells to stop bleeding. Wu et al. [90] found that ionic self-assembly of nanopeptide exhibited a dual effect of rapid hemostasis and accelerated osteosis. Most protein materials play a role in the form of covalent binding modification, among which collagen is widely used in dental surgery. Various collagen-based biomaterials have been reported to have the ability to simultaneously stop bleeding and promote bone regeneration [91,92].

### Synthetic polymers

**Fig. 5.**
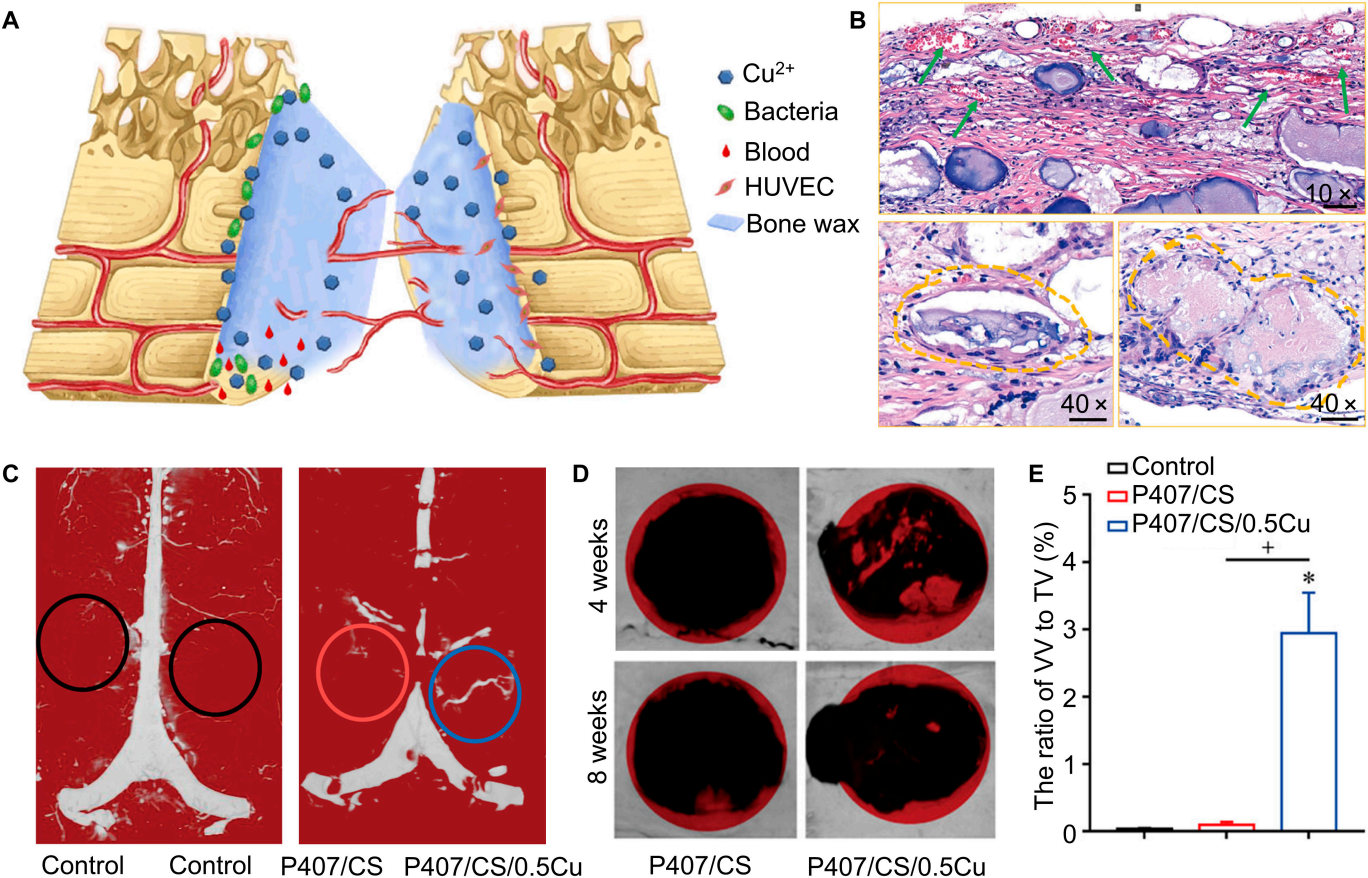
Examples of bone hemostatic materials based on synthetic polymers. (A) Conceptual diagram of the feasibility of applying materials to the defective bone. (B) Histology section hematoxylin and eosin (H&E) staining after 4 weeks of implantation of P407/CS/0.5Cu at the bone defect. Green arrows mark neovascularization while yellow dotted circles mark residual bone wax and aggregated macrophages. (C) to (E) are angiogenesis experiments in vivo. The corresponding materials were given to the rat model of a skull defect, and the vascular growth in the bone defect area was observed after 8 weeks. (C) Micro-CT angiography images. (D) Micro-CT images. (E) The ratio of in vivo vascular volume to tissue volume. Reproduced with permission from Ref. [94]. Copyright 2021 American Chemical Society.

Synthetic polymers, possessing adjustable physical and chemical properties, play a key role in bone hemostatic and bone regeneration. Bone wax is a classic bone hemostatic material in the clinic, whereas its drawbacks have hampered its application. Therefore, it is urgent to develop new bone waxes that can biodegrade, coexist with the body, inhibit the growth of bacteria, and promote the growth of blood vessels. Poloxamer, having a trading name “Pluronic”, is a unique material, which is in a gel state at room temperature and a liquid state at low temperature [93]. Yan et al. [94] developed a biodegradable bone wax made from a mixture of calcium sulfate, Pluronic 407, and copper ions, as shown in Fig. 5. Unlike traditional bone waxes, this copper-loaded biodegradable bone wax is biocompatible, having the ability to promote angiogenesis, resist infection through the release of copper ions, and improve the microenvironment at the fracture site. In addition, Pluronic can promote cartilage tissue formation, facilitate the vascularization of injured bone tissue, and protect periodontal bone [95,96]. BoneStat, a newly developed bone hemostatic material, has the advantages of promoting hemostasis, simple operation, and autonomous degradation [97]. Polyethylene glycol (PEG) is also representative of traditional hemostatic materials. Brückner et al. [9] have developed a new type of self-coagulating bone wax prepared based on PEG, inorganic non-metallic materials, and starch, which not only has ductility but also has certain hardness and drug-carrying characteristics after hardening. Other studies found that PEG can directly or indirectly promote angiogenesis, protect local tissue to fight against bacteria, and exhibit perfect flexibility [98]. Compared with commercial hemostatic materials such as Ostene and BoneSeal, these new hemostatic materials have made more or less material or technical improvements to cope with more changing needs [99].

### Inorganic materials

**Fig. 6.**
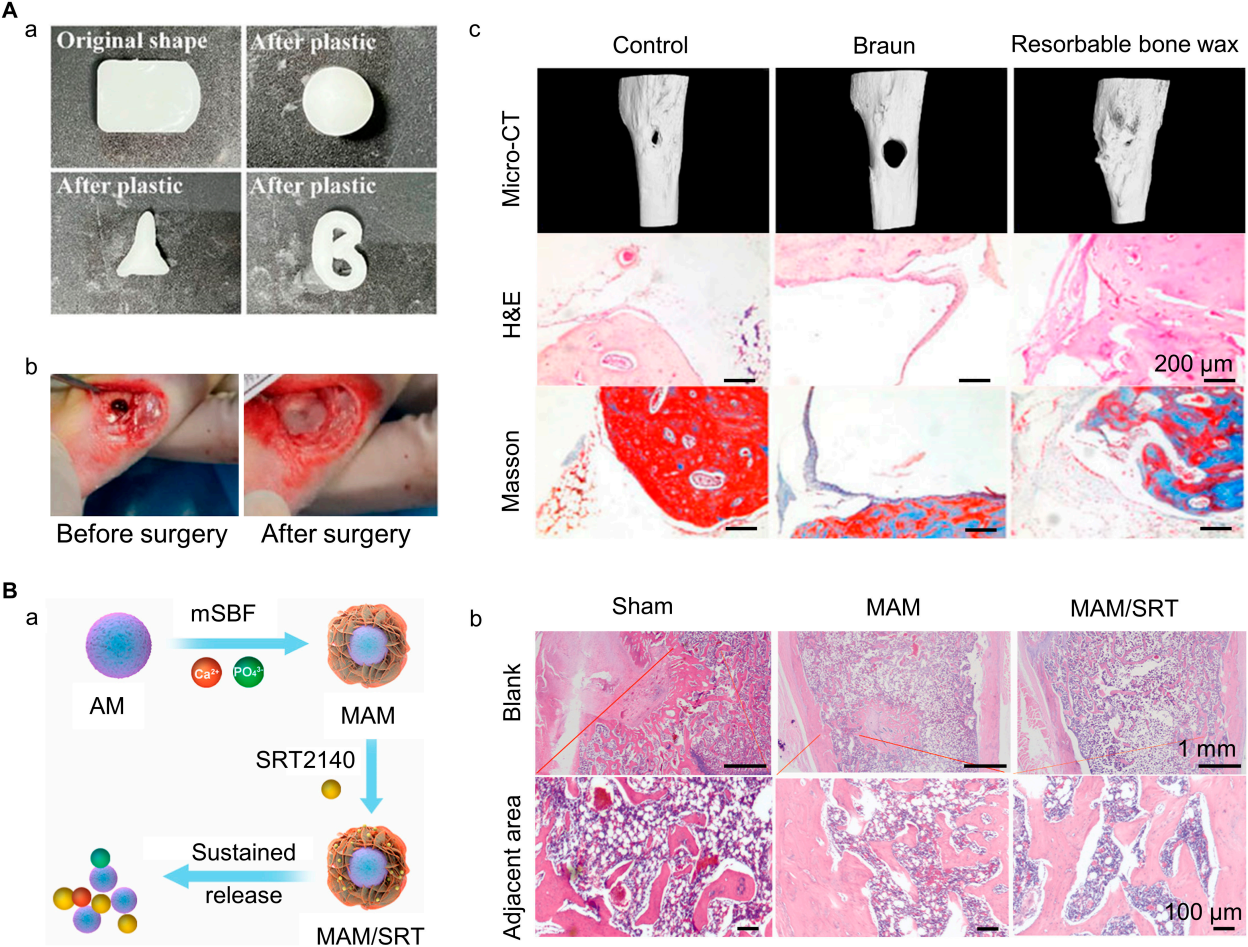
Examples of inorganic hemostatic material. (A) A novel β-TCP absorbable bone wax that promotes bone hemostasis and bone regeneration. (a) Plasticity of the novel bone wax. (b) Therapeutic status of the novel bone wax. (c) Images of the state of the tibia 6 weeks after injury. Reproduced with permission from Ref. [70]. Copyright 2023 Duan, Liu, Zheng, Cai, Huang, Liu, and Guo. (B) Bone healing of osteoporotic bone: (a) Schematic diagram of the action of mineral particles. Mineral coating cell-free matrix particles loaded with SRT2104, which regulates the state of osteoblasts and osteoclasts by continuous release at the site of injury, reversing osteoplasts. (b) H&E staining of the area of rat femoral injury in the fifth week after surgery. Reproduced with permission from Ref. [106]. Copyright 2022 The Authors.

Inorganic non-metallic materials are mineral-based materials, which promote hemostasis by absorbing plasma to concentrate blood. Some inorganic materials containing calcium or phosphorus elements can release bioactive metal ions in the physiological environment to promote bone tissue regeneration [100,101]. In the section, bone wax, bone cement, and bioceramics are introduced as typical inorganic hemostatic materials. Duan et al. [70] designed a novel type of absorbable bone wax that overcame the shortcomings of traditional bone wax. This bone wax is based on epoxyane copolymer and additionally added β-tricalcium phosphate (β-TCP) and starch microspheres to enhance its ability to stop bleeding and promote bone regeneration. In the investigation of the rabbit tibial defect, compared with classical bone wax, it can quickly stop bleeding and rapidly degrade (within 6 weeks), which is conducive to tissue regeneration (Fig. 6A).

Bioactive ceramics can directly act on the body, making cell tissues grow into it through interaction with the body [102]. Mesoporous bioactive glass (MBG), a new generation of nanostructured glass, is widely used due to its superior surface texture, porosity, and bioactivity. For instance, an MBG doped with bismuth oxide (Bi_2_O_3_) was synthesized by a 2-step acid-catalyzed self-assembly process [103]. By optimizing the doping concentration of Bi_2_O_3_, MBG can achieve a specific surface area as high as 704.06 m^2^/g. Compared to other groups, Bi_2_O_3_-doped MBG can accelerate coagulation reaction by causing platelet aggregation and promoting fibrin polymerization. However, an excessive amount of Bi_2_O_3_ is counterproductive, hindering the coagulation effect. Hydroxyapatite (HAP), which is the main inorganic component of natural bone, is also widely utilized in bone tissue engineering and hemostatic application due to its good biocompatibility, bone induction, and ability to adsorb proteins and form blood clotting due to the surface charge. Ma et al. [104] prepared a temperature-responsive multifunctional nano-hydroxyapatite/graphene oxide/chitosan (nHAP/GO/CS) scaffold. Under the irradiation of 808-nm near-infrared light, it can quickly stop bleeding and coordinate human bone marrow mesenchymal stem cell osteogenesis when the temperature reaches around 42 °C. Considering the diversity of bone defect shapes, 3D bioprinting technology provides a personalized design of active scaffolds to repair and reconstruct the defects of weight-bearing bone [105].

Osteoblasts and osteoclasts in the bone injury area are active, and once the local bone homeostasis is broken, it is easy to cause problems such as bone dysplasia and osteoporosis. Therefore, it is important to adjust the balance of the two as needed. Zhang et al. [106] have realized a new molecular therapy that can be used to improve bone homeostasis imbalance. The mineral-coated cell-free matrix particles are compounded SRT2104 and continuously released at the site of injury, ultimately regulating osteoblast and osteoclast production, thereby reversing bone homeostasis imbalance. The osteoporosis rat model verified that this method can accelerate the healing of osteoporotic bone and help bone remodeling (Fig. 6B). Li et al. [107] designed an n-HAP/resveratrol/CS composite microsphere for bone crisp pine. The spheroids have anti-inflammatory activity and promote hemostasis and healing of femoral defects in rat models of osteoporosis.

### Nanocomposite materials

**Fig. 7.**
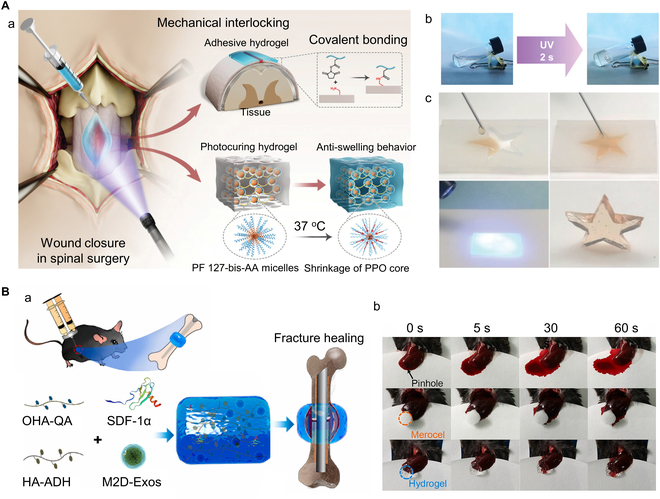
Examples of composite bone hemostatic materials. (A) RAAS hydrogel: (a) Conceptual diagram of RAAS hydrogel. The hydrogel has fast adhesion to wet tissues and anti-swelling properties. Therefore, it can quickly stop bleeding from wounds and reduce the compression of tissues and organs, such as the spinal cord and internal organs, around deep wounds. (b) Cross-linking of RAAS hydrogel with ultraviolet light. (c) Injectability and curability of RAAS hydrogels. Reproduced with permission from Ref. [108]. Copyright 2022 Wiley-VCH GmbH. (B) An HA-based versatile hydrogel that combines stromal cell-derived factor-1α (SDF-1α) and exosomes (M2D-Exos): (a) Schematic diagram of the HA@SDF-1α/M2D-Exos hydrogel, which has antibacterial and hemostatic effects and can promote bone growth and neovascularization. (b) Liver bleeding experiments are performed to test the hemostatic performance of the merocel (a hemostatic sponge) and the prepared composite hydrogel. Reproduced with permission from Ref. [109]. Copyright 2022 The Authors.

For a single material, it is hard to meet the requirements of functionality and application. In contrast, nanocomposite materials combine 2 or more materials, inheriting the advantages of each component and even presenting novel characteristics. Hence, nanocomposite materials have attracted more and more attention. For instance, Bian et al. [108] used heat-sensitive shrinkable micellar gels and small-molecule binders to co-produce an injectable hydrogel (named RAAS hydrogel) for rapid adhesion and anti-hygroscopic expansion in wet tissues (Fig. 7A). The hydrogel can form gel rapidly on demand, and adhere to the wound to stop bleeding in time. Chen et al. [109] combined stromal cell-derived factor 1α (SDF-1α) and M2 macrophage-derived exosomes (M2D-Exos) with HA-based hydrogel precursor solutions to create HA@SDF-1α/M2D-Exos hydrogels, as shown in Fig. 7B. The hydrogel is injectable, self-healing, and infection-resistant, and rapidly stops bleeding, promoting the healing of bone tissue by promoting bone regeneration and angiogenesis.

The application potentials of various nanocomposite materials in bone hemostasis and bone repair have been investigated. An inorganic–organic hybrid hydrogel based on silk fibroin has been developed, which has a self-healing ability similar to natural bone tissue. It can also withstand a large amount of mechanical strength and support bone tissue regeneration at murine femoral defects [110]. There is an acid-responsive composite hydrogel scaffold using multi-style F127 diacrylate as the base scaffold material and nano-CaCO_3_ as the composite material. The hydrogel scaffold promotes bone regeneration in the rabbit skull by supplying calcium ions and provides controlled mechanical strength [111]. A BG/CS/carboxymethylcellulose composite scaffold was designed to replace the traditional bone wax in the clinic, which also has the characteristics of promoting bone regeneration and timely biodegradation in damaged areas [112].

## Conclusion and Future Outlook

Bone hemostatic biomaterials can help the damaged areas to stop bleeding quickly and provide a platform for the morphological and functional recovery of tissues at these damaged areas. In terms of selection and design of suitable materials, the compositions, the characteristics (e.g., morphology, shape, inner structure, and mechanical strength), the performance (e.g., biocompatibility, biodegradability, and capacity of osteogenesis and hemostasis), and the location of the wound should be collectively taken into consideration. Understanding the hemostatic mechanism and the instinct characteristics of various materials can promote the development of bone hemostatic biomaterials from the bench to the bedside.

So far, most hemostatic materials have been applied to the major tissues such as skin, liver, and kidneys. There are relatively few reports on bone hemostasis. Regarding the development of bone hemostasis in the future, it is recommended to design intelligent biomaterials, which can deliver bioactive particles, drugs, or functional factors to the targeted wound site regardless of the size and depth of the wound site and release these therapeutic agents on demand (e.g., upon real-time stimuli such as temperature, light, pH, enzymes, and signaling proteins). Furthermore, it would be better if the bioactive materials have the capacity of shape transformation to adapt to various bone defects and the changing physiological environment in a timely manner. In addition to blood vessels and bones, the reconstruction and restoration of nerves are also worthy of investigation. In more challenging neural tissue studies, the research on the role of electrical stimulation and conductive hemostatic materials in the wound healing process is still limited [113]. Hence, a comprehensive set of experimental methods and standards needs to be explored to explore neurological-related problems in the bone healing process.

Regarding bone regeneration, the materials mainly for hydrogels need to obtain almost all the characteristics of bone hemostatic materials above. Besides, successful bone regeneration also requires coordination between cells, growth factors, and the backbone of hydrogel materials. Bone healing sites mostly lack neovascularization, and hence, the incorporation of angiogenic factors into hydrogels is an effective strategy to promote bone repair and regeneration. It is also possible to promote angiogenesis by directly adding cells associated with angiogenesis or modifying the function of hydrogel materials. However, the treatment efficiency of this method is still not that ideal because many directly added biofactors may have other limitations such as instability, inactivation, or decomposition. Therefore, future treatment modalities for angiogenic factors can be treated by attaching antioxidants and other stabilizers, or by targeted release and transport, thereby minimizing the unnecessary loss and maximizing the bioavailability.

The hematoma is an important stage of bone healing. In the early stage of bone fracture, blood vessels and soft tissues are destroyed, which make macrophages and platelets release cytokines to recruit stem cells to the bone healing site, inducing hematoma and inflammation. However, the content of blood cells, immune cells, and pro-inflammatory factors in the hematoma may cause a relatively large burden, which corresponds to the urgent repair state of bone tissue in the hematoma environment, and larger hematomas may result in potential risks for nerve compression. Whether it is a bone hemostatic material or a bone regeneration material, research progress revealed that the local microenvironment, especially the immune microenvironment, plays a crucial role in bone hemostasis, healing, and regeneration, which requires different subpopulations of immune cells to promote development throughout the process of bone regeneration [114]. During the bone repair process, the initially formed pro-inflammatory immune microenvironment, mainly induced by M1 macrophages, is beneficial for immune cells to remove pathogens and cell debris and maintain local stability of the wound [115]. However, the excessive inflammatory response may hinder the bone tissue repair, thus gradually transforming the pro-inflammatory immune microenvironment into an anti-inflammatory immune microenvironment mainly induced by M2 macrophages [116], which facilitates tissue cell proliferation and regeneration. In addition, other immune cells are also involved in the regulation of bone healing, such as dendritic cells that can regulate early bone tissue healing activities and inhibit mineralization [117]. T cells can promote bone tissue regeneration and osteogenesis; however, an excessive content of CD8 T cells can damage bone tissue, causing a disappointing repair [118,119]. Therefore, immune cells play crucial roles in bone-related physiological and pathological processes. By appropriately designing bioactive materials to regulate the activities of immune cells to achieve the required immune microenvironment of bone tissue, bone tissue repair can be effectively promoted and the existing treatment strategies can be improved accordingly, which will become the a promising research hotspot in the future.

Collectively and ideally, biomaterials for bone hemostasis and bone regeneration should possess the following characteristics: rapid hemostasis and healing [120], biocompatibility [58], biodegradability [79], suitable mechanical strength [110], antibacterial capacity [121], and capacity of promoting bone regeneration and repair [85]. To modulate the microenvironment, it is worth noting that biomaterials are supposed to modulate inflammatory response on demand. In the early stage of healing, a proper inflammatory response is required, and the tissue cells in the inflammatory area can secrete a sufficient number of pro-inflammatory factors to attract inflammatory cells with the local dilating capillaries and the increasing temperature, which is conducive to the repair of the initial stage of inflammation. However, in the middle and late stages of healing, the intense inflammatory response is not conducive to the development of repair, and even it is harmful for normal tissue cells. Therefore, the design of biomaterials needs to target the inflammatory response at the appropriate time. To meet surgical requirements and provide continuous protection for human health, it is imperative to design and develop novel bone hemostatic materials for bone tissue engineering.

## Data Availability

Data of this paper are available by emailing lijinhua@bit.edu.cn.
